# Similarities and differences in the late-onset GM2 gangliosidoses: Tay-Sachs and Sandhoff diseases

**DOI:** 10.1007/s00415-026-14034-2

**Published:** 2026-08-03

**Authors:** Connor J. Lewis, Leila Shirvan, Jean M. Johnston, Catherine Groden, John Yang, Andrea Ashton, Jessica Chong, Mark Moran, Hera Akmal, Selby I. Chipman, Cris Zampieri, Jordan Wickstrom, Jesse Matsubara, Tanya Lehky, Katharine E. Alter, Camilo Toro, Cynthia J. Tifft

**Affiliations:** 1 Office of the Clinical Director, National Human Genome Research Institute, Bethesda, MD 20892 USA; 2 Medical Genetics Branch, National Human Genome Research Institute, Bethesda, MD 20892 USA; 3 Neurorehabilitation and Biomechanics Research Section, National Institutes of Health Clinical Center, Bethesda, MD USA; 4 ABBEL Research Division, Rehabilitation Institute at Sinai (Formerly Sinai Rehabilitation Center), Sinai Hospital, Baltimore, MD USA; 5 Electromyography Section, National Institute of Neurological Disorders and Stroke, Bethesda, MD USA

**Keywords:** Tay-Sachs, Sandhoff, GM2 gangliosidosis, Lysosomal storage disease

## Abstract

Due to shared ß-hexosamindase A deficiencies and significant clinical overlap, the predominating subtypes of late-onset GM2 gangliosidosis, late-onset Tay-Sachs (LOTS) and late-onset Sandhoff disease (LOSD) have been considered essentially indistinguishable. However, growing evidence supports several distinctions between the two entities. We highlight these distinctions through the cross-sectional evaluation of 27 (21 LOTS and 6 LOSD) late-onset GM2 gangliosidosis participants. Study protocol included physical examinations, assessments of gait, balance, muscle strength, ataxia, and nerve conduction velocities, and brain magnetic resonance imaging. Lower limb weakness and later development of upper limb weakness was highly prevalent in both diseases. Accompanying gait disturbances, balance issues, and dysmetria were also prevalent in both cohorts. Strength evaluations showed weakness in both the LOTS and LOSD cohorts compared to controls primarily impacting extensor muscles. In contrast, BARS scores for dysarthria and oculomotor dysfunction were present and heterogenous in LOTS participants and absent in LOSD participants. Twenty-four percent of LOTS participants and none of the LOSD participants had a history of neuropsychiatric symptoms. Cerebellar volume including lobules V and VI were lower in LOTS compared to LOSD and normative data. However, severe length-dependent sensory neuropathy was present in all LOSD participants but not in LOTS participants. The finding of lower cerebellar volume in LOTS suggests the distinctive features of the LOTS phenotype are related to cerebellar dysfunction. However, the cause of the phenotypic differences between LOTS and LOSD remains a mystery, and the molecular and biochemical basis for the dichotomy requires further investigation.

**Supplementary Information:**

The online version contains supplementary material available at 10.1007/s00415-026-14034-2.

## Introduction

The GM2 gangliosidoses are rare autosomal recessive lysosomal storage disorders caused by the accumulation of GM2 ganglioside due to the absence or near absence of ß-hexosamindase A (EC 3.1.2.52), the heterodimeric enzyme that degrades glycosphingolipid GM2 in healthy tissue of unaffected individuals. Pathological accumulation of GM2 ganglioside unleashes a cascade of events leading to progressive neurodegeneration [1,2]. Tay-Sachs and Sandhoff diseases are the predominate forms of GM2 gangliosidosis with a prevalence of 1 in 200,000–320,000 and 1:500,000–1,500,000 individuals, respectively [3–5] An additional ultrarare form caused by biallelic variants in *GM2A*, encodes the GM2, the activator protein required for GM2 degradation [6]. The prevalence of Tay-Sachs disease is higher among the Ashkenazi Jewish and non-Jewish French Canadians from southeastern Quebec due to founder effects [5, 7–9]. Tay-Sachs disease (OMIM #272800) is caused by biallelic pathogenic mutations in *HEXA*, encoding the alpha subunit of ß-hexosaminidase A, while Sandhoff disease (OMIM #268800) is caused by biallelic pathogenic mutations in *HEXB*, which encodes the beta subunit of ß-hexosaminidase A [2, 10,11].

Both Tay-Sachs and Sandhoff diseases are a continuum of disease severity classified into infantile, juvenile, and late-onset (adult) forms based on the timing of symptom onset approximately correlated with residual ß-hexosaminidase A activity [12,13]. The infantile forms of these diseases are the most severe with symptom onset occurring before six months and death by five years of age [14,15]. The juvenile form of these diseases is less severe with symptom onset between 2 and 6 years of age and death resulting within the second decade. The adult or late-onset form of Tay-Sachs or Sandhoff diseases are the most heterogeneous, with symptom onset beginning during adolescence or early adulthood and life expectancy slightly reduced compared to unaffected individuals [16–18]. There is no targeted treatment for the GM2 gangliosidoses; however, trials of gene therapy and the modified amino acid *N*-acetyl-L-leucine (NALL) have been recently reported [19,20].

Patients with late-onset GM2 present with neuromuscular weakness, cerebellar dysfunction, and/or psychiatric symptoms [17,18]. There is substantial heterogeneity in presenting symptoms and disease progression of patients, even among siblings sharing the same mutations and genetic background [21–23]. Neurodegeneration is inexorably progressive over decades with worsening weakness, incoordination, dysarthria, and, in the later stages of the disease, cognitive dysfunction [18, 24,25]. Late-onset Tay-Sachs (LOTS) and late-onset Sandhoff disease (LOSD) have long been considered clinically identical. However, some phenotypic features of LOTS and LOSD appear more prevalent in one form over the other. For example, psychosis is more characteristic of LOTS while early sensory symptoms seem to be more pronounced in LOSD [17]. Pure cerebellar dysfunction and dysarthria may be more severe in LOTS [24,25]. However, weakness and ambulation difficulties are common to both [18]. Strength testing on physical examination shows earlier involvement in the lower limbs, more specifically in the hip flexor and knee extensor muscles with later progression to the upper limbs, involving primarily the triceps. Electrophysiological studies indicate neurogenic weakness consistent with an anterior horn neuronopathy, a shared phenotype of LOTS and LOSD [10].

Magnetic resonance imaging (MRI) studies have demonstrated numerous findings at different stages of the disease [10, 26], however cerebellar atrophy with associated enlargement of the 4th ventricle is reported in both LOTS and LOSD [27,28]. One study, including only participants with LOTS, analyzed specific lobules of the cerebellum and suggested a link between atrophy in cerebellar lobule VI to ataxia, dysarthria, and tremors [29]. Cerebellar atrophy may be more common in LOTS compared to LOSD [28]. Due to the slow progression of the disease, capturing clinically reliable metrics for disease progression has been difficult. A study evaluating potential clinical outcomes, suggested the nine-hole peg test (9HP), the Friedreich’s Ataxia rating scale (FARS), and the assessment of intelligibility of dysarthric speech (AIDS) were promising tests for assessing disease progression [30].

In this study, we conducted cross-sectional evaluations in a cohort of LOTS (*n* = 21) and LOSD (*n* = 6) participants enrolled in a natural history study. Specifically, we collected data from instrumented gait and balance analysis, lower limb (knee flexor and extensor) muscle strength dynamometry, nerve conduction velocities (NCVs) and needle electromyography (EMG), speech intelligibility, and brain MRI anatomical, volumetric, and advanced imaging analysis. This comprehensive dataset was intended to capture the nature and spectrum of functional impairment in late-onset GM2 disease, explore phenotypic differences between LOTS and LOSD, and correlate aspects of the clinical history and presenting symptoms to additional disease phenotypes.

## Methods

### Participants

Participants diagnosed with LOTS or LOSD were enrolled at the National Institutes of Health (NIH) Clinical Center under the “Natural History of Glycosphingolipid and Glycoprotein Disorders” (ClinicalTrials.gov identifier: NCT00029965). [31]. The NIH Institutional Review Board approved this protocol (02-HG-0107). Informed consent was completed with all participants prior to participation, and all research was completed in accordance with the Declaration of Helsinki. Participants were evaluated between May 2012 and July 2025 at the National Institutes of Health Clinical Center in Bethesda, Maryland. Participants underwent a variety of clinical evaluations including a physical examination, gait analysis, balance testing, dynamometry strength testing, electrodiagnostic investigation, speech, and magnetic resonance imaging (MRI). All LOTS and LOSD participants with at least one of the aforementioned evaluations enrolled in NCT00029965 were included in this study. Although the order of testing (functional testing, strength, balance, and gait) varied across participants, a rest period of at least 20 min was provided between testing sessions to allow participants to recover from possible fatigue. For participants with repeated evaluations, the visit with the most complete data in terms of testing was selected. Demographics and medical histories were reported using a questionnaire modified from Bley and colleagues [32]. Some participants were assisted by a family member due to difficulty with writing or inability to recall details of their disease progression. Diagnosis was confirmed by enzyme analysis in peripheral blood leukocytes and/or mutation analysis of *HEXA* and *HEXB* genes performed in CLIA-certified laboratories. 21 LOTS and 6 LOSD participants were included and further information on the participants are provided in Table 1 and Supplement Table 1. Some participants did not complete all the tests described in this study because of personal choice or safety concerns.

**Table 1:** Summary of Participant Characteristics

	Participant characteristics									Symptoms			
ID#	Diagnosis (LOTS/LOSD)	Age (Yr)	Age at Handrails^a^	Age at Diagnosis		Sex	Initial Presenting Symptom^b^	Lower Limb Weakness (±)	Upper Limb Weakness (±)	Dysphagia (±)	Dysarthria (±)	Tremor^c^	Major Psychiatric Events (±)
1	LOTS	27	17	20		F	F	+	+	+	+	PO, E	–
2	LOTS	37	13	32		F	F	+	+	–	–	PO	–
3	LOTS	45	18	27		F	W, Sp	+	+	+	+	K	–
4	LOTS	53	45	50		M	W	+	+	–	+	PA	–
5	LOTS	27	18	21		F	W	+	+	–	–	PO, E	–
6	LOTS	52	35	45		M	W	+	+	–	–	PA	–
7	LOTS	28	20	26		F	N/A	+	+	–	+	PO	–
8	LOTS	28	18	26		F	T, W	+	+	–	+	PO	–
9	LOTS	59	40	53		F	W, F, T	+	+	–	+	–	–
10	LOTS	33	18	21		M	P	+	+	–	+	PO, E	+
11	LOTS	31	21	18		F	N/A	+	+	–	+	PO, E	+
12	LOTS	37	32	34		M	W, F, Sp	+	+	–	+	PO, K	–
13	LOTS	23	20	20		M	N/A	+	+	–	+	PO	–
14	LOTS	33	28	30		M	N/A	+	+	+	+	PO, K	–
15	LOTS	45	32	41		M	W, T, P	+	–	+	–	K	+
16	LOTS	46	28	42		M	F	+	+	–	+	K	–
17	LOTS	35	18	29		F	N/A	+	+	–	–	PO, E	–
18	LOTS	35	20	33		M	W, F	+	+	+	+	PA, PO	–
19	LOTS	23	N/A^a^	0^d^		F	N/A	+	+	–	+	E	+
20	LOTS	42	N/A^a^	34		F	W, Sp	–	–	–	+	K	+
21	LOTS	70	29	67		F	F, W	+	+	–	–	–	–
22	LOSD	62	17	58		F	N/A	+	–	+	–	PO	–
23	LOSD	51	23	48		M	W, Se	+	+	–	–	–	–
24	LOSD	58	17	56		M	W,F,T	+	+	–	–	PO	–
25	LOSD	56	46	54		M	N/A	+	+	–	–	PO	–
26	LOSD	44	39	40		F	W	+	+	–	–	–	–
27	LOSD	53	35	53		F	W	+	+	–	–	PO	–

^a^N/A = Participants did not require handrails at the cross-sectional evaluation

^b^*F* falling, *W* weakness, *Sp* speech, *T *tremor, *P* psychiatric event, *Se* sensory symptoms, *N/A* diagnosed based on sibling

^c^*PO* postural tremor, *E* endpoint tremor, *K* kinetic tremor, *PA* parkinsonian tremor,—= no tremor

^d^Participants diagnosed in utero

### Functional testing

Functional testing was performed by the same research nurse (JMJ) for all participants. Functional testing included the Rolyan 9-Hole Peg (9HP) test [33], The Timed Get Up and Go Test (TGUG) [34], and Trail Making Test Part A and B (TMT-A/B) [35,36]. The 9HP test was performed twice bilaterally, and the times (in seconds) from the dominant hand were averaged and reported. The TGUG test was performed with the participant sitting in a neutral position in an armchair, and the time (in seconds) required to stand up, walk 10 feet, and return to the chair were recorded in seconds. TMT-A and TMT-B were performed using the participant’s dominant hand.

### Ataxia

Ataxia was evaluated using the brief ataxia rating scale (BARS) by a qualified neurologist (CT) [37]. The complete BARS is scored out of 30, with five domains including gait (8 points), knee-tibia test (4 points for each side), finger-to-nose test (4 points for each side), dysarthria (4 points), and oculomotor abnormalities (2 points). When the examiner could not commit between two scores on a BARS item, an intermediate score was provided.

### Gait analysis

Gait was evaluated using the 6-min walk test. Participants walked in a quiet, well-lit hospital hallway at their preferred pace for six minutes. They were instructed to walk close to the railing on one side of the corridor and perform turns when they reached the end of a linear, 25-m walking path. A physical therapist walked behind the participant for safety. To avoid falls due to fatigue, participants were reminded that they were not required to complete the task and could stop at any time. A 4.8-m instrumented walkway (GAITRite; CIR Systems Inc., Sparta, NJ) was placed within the walking path [38,39]. The walkway allowed for capturing the participant’s steady state gait (avoiding the acceleration and deceleration gait phases at the ends of the walking path). The footprints for each pass on the instrumented walkway were analyzed for gait speed (centimeters per second), step length (centimeters), step width (centimeters), and double support time (seconds). All passes were averaged as well as right and left sides for analysis of each parameter. Age and sex matched controls were extracted from McKay et al. [40]

### Balance testing

Study participants performed the Modified Clinical Test of Sensory Interaction on Balance (mCTSIB) as described in Antoniadou et al., [41], on a NeuroCom Balance SMART Equitest® System (previously Natus Medical Inc., Seattle, OR, USA). Participants stood barefoot in quiet stance for 10 s trials. Each trial was performed 3 times under each of the following standing conditions in the following order: firm surface with eyes open, firm surface with eyes closed, foam surface with eyes open, and foam surface with eyes closed. A given condition was not tested if the participant was unable to stand barefoot on the surface or if the test was perceived as unsafe. The sway velocity of the center of gravity in degrees/second was recorded from each trial. An average sway velocity of 3 trials for each condition was used in the data analysis. Higher sway velocity scores indicate poorer postural control. Scores reaching a maximum of 6 degrees/second indicated a fall. For participants who were unable to complete all trials due to safety concerns, a value of 6 degrees per second (worst possible score) was assigned. Age and sex matched controls were obtained directly from NeuroCom Balance Systems Customer Support.

### Dynamometry strength of quadriceps and hamstring muscles

A Biodex System 3 (Shirley, NY) was used to measure isometric and isokinetic knee extensor (quadriceps) and flexor (hamstring) strength. Participants were seated in the Biodex chair with the knee joint aligned along the dynamometer axis. The leg was secured with a strap across the thigh and the dynamometer arm secured near the malleoli. Participants completed 3 trials of isometric knee flexion and extension for 5 s separated by 15 s rests to allow for recovery. Knee flexion trials were measured starting from the participant’s maximum extension, and knee extension trials starting from the participant’s maximum flexion.

Participants completed 3 self-paced isokinetic flexion and extension trials at 60 degrees/second and a 30 s rest period between flexion and extension trials. Flexion was tested from participants’ end range knee extension while seated in the Biodex to the maximum flexion angle that could be achieved before the dynamometer arm contacted the Biodex chair. Extension was tested from the knee flexed, with the dynamometer arm against the Biodex chair, to participants’ end range knee extension. The maximum force in Newtons (N) during each trial was recorded and converted to Torque (Nm). The average of the three trials was calculated and normalized in relation to the participant’s weight in kilograms (Nm/kg). Control data were obtained from healthy volunteers recruited by the Rehabilitation Medicine Department of the Clinical Center, NIH.

### Sensory examination and electrodiagnostic testing

A bedside proprioceptive sensory exam was performed in all individuals as part of their clinical examination. This included a measure of the time-to-extinction of vibration perception after of application of the stem tip of a fully activated “C” 128 Hz tuning fork the base of the great toe. Participants scored normal (> 15 s), mildly decreased (10–15 s), severely decreased (< 10 s), or absent. Nerve conduction studies were performed using standard methodology on a Natus Viking Select or EDX machine (Natus, Middleton, WI). In general, sensory nerves (e.g., median, sural) and motor nerves (e.g., fibular, median) were tested, though the specific selection of nerves depended on clinical status. Needle EMG was performed using a concentric needle and filter settings of 2 k–10 k with spontaneous and motor unit potential activity recorded according to standard methodology. Choice of muscle for needle EMG studies was dependent on the distribution of weakness. Active denervation changes in this study were marked by the presence of fibrillation potentials and positive sharp waves (fibs/psw) with or without the presence of complex repetitive discharges. Chronic neurogenic changes in this study were marked by the presence of long duration and/or high amplitude motor unit potentials with possible polyphasia. Generally, decreased recruitment was observed in the presence of neurogenic changes. Parameters were compared to department-based normative data rather than explicit controls.

### MRI

As described in Lewis et al. [27], T1-weighted magnetic resonance imaging (MRI) from 23 GM2 Natural History participants were acquired on a Phillips Achieva 3 T system with an 8-channel SENSE head coil. 18 LOTS participants and 5 LOSD had MRI imaging at the cross-sectional visit selected. Volumetric analysis of MRI data was performed using Freesurfer’s (v7.4.1) *recon-all* reconstruction pipeline to calculate volumes of the gray matter, white matter, cerebellum, 4th ventricles, and total intracranial volume (ICV). and thalamus [27, 42–50]. One thousand thirty-three neurotypical controls (NC) were also analyzed from OpenNeuro and included participants from the following studies: “The National Institute of Mental Health (NIMH) Intramural Healthy Volunteer Dataset” (*n* = 154) [51,52], “Neurocognitive aging data release with behavioral, structural, and multi-echo functional MRI measures” (*n* = 298) [53,54], “Paingen_placebo” (*n* = 395) [55,56], and “AgeRisk” (*n* = 186) [57,58] consisting of participants between 18 and 89 years old. T1-weighted MRI were also analyzed at the cerebellar lobule level as described in Lewis et al. to calculate volumes of lobules V and VI. [59]

### Statistical analysis

Due to the small sample size, particularly in the LOSD cohort, data was descriptive in nature. Frequencies were used to summarize categorical clinical symptom data. Ordinal BARS scores were presented as medians and inner quartile ranges. Otherwise, when appropriate, data are reported as means (± SD). Data analysis and graph generation were performed in GraphPad Prism software for macOS, version 10.1.0 (GraphPad Software).

## Results

### Participants and clinical description

Twenty-one participants with LOTS (from 16 families, 12 females) and 6 participants with LOSD (from 4 families, 3 females) were evaluated under this protocol. Demographic data are presented in Table 1 and more extensively described in Supplement Table 1. At the time of this cross-sectional evaluation, physical examination revealed the presence of lower limb weakness in 20 LOTS (95%) and 6 LOSD participants (100%) and upper limb weakness in 19 LOTS (90%) and 5 LOSD participants (83%). All participants from both groups had muscle atrophy. Cerebellar dysfunction was present in 20 LOTS participants (95%) and 3 LOSD participants (50%). Dysphagia was present in 5 LOTS participants (24%) and 1 LOSD participant (17%), 15 LOTS participants (71%) and no LOSD had dysarthria, 5 LOTS participants (24%) and no LOSD participants had a history of a major psychiatric event. No LOTS participants and all 6 LOSD participants had evidence of sensory neuropathy and dysautonomia (Table 1, Supplement Table 1).

### Symptom onset

**Fig. 1:**
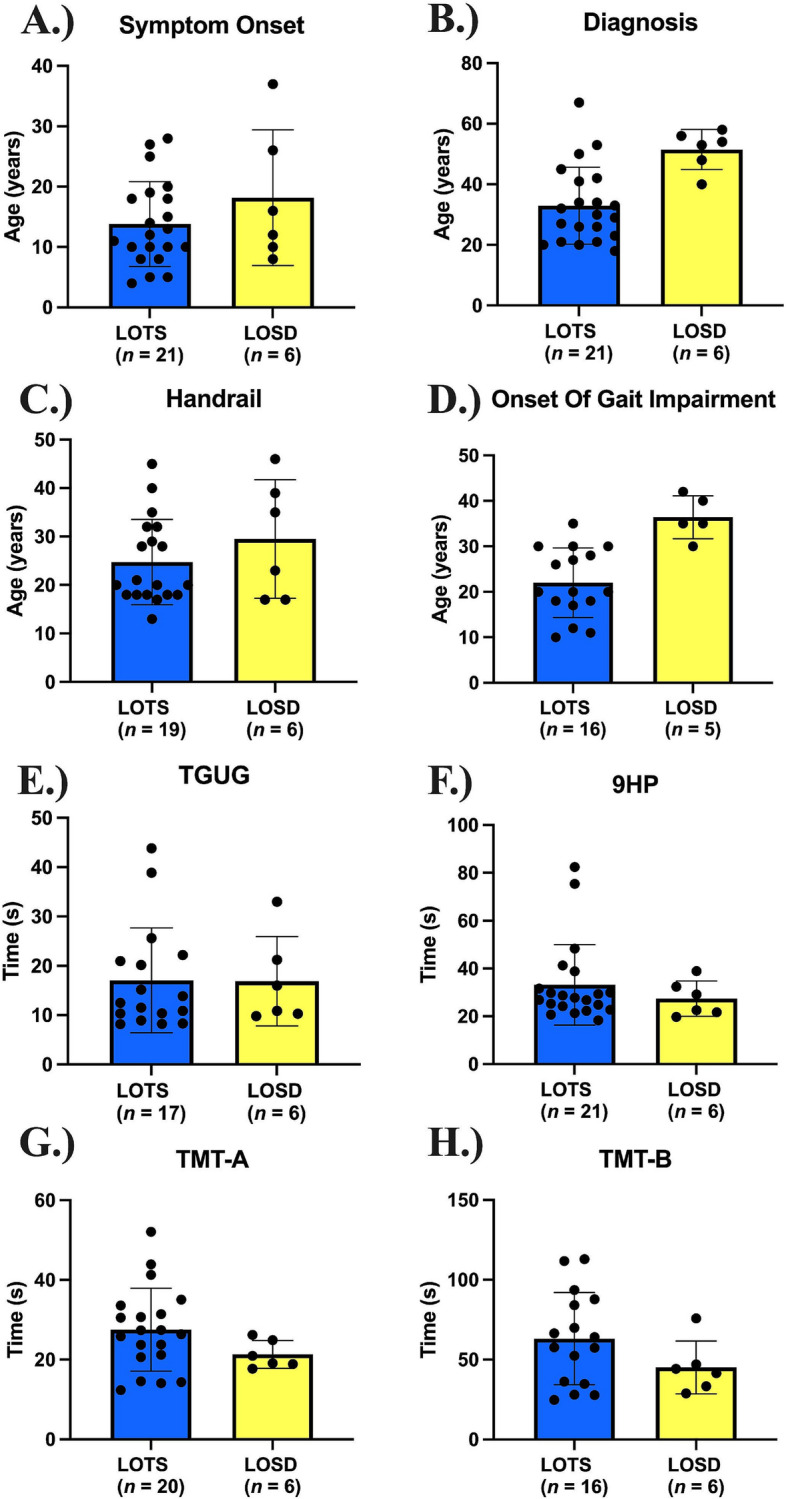
Clinical Phenotype and Clinical Outcome Assessments. **A** Age of Symptom onset between LOTS and LOSD. **B** Age at diagnosis between LOTS and LOSD. **C** Age when Handrails were required. **D** Age at the onset of gait impairment. **E** Time get up and go (TGUG) functional testing. **F** 9-Hole Peg (9HP) Test on the dominant hand. **G** Trail Making Test (TMT) Part A. **H** Trail Making Test Part B

Participant reported symptom onset in the LOTS and LOSD cohorts occurred at an average age of 13.8 ± 7.0 years and 18.2 ± 11.2 years, respectively (Fig. 1A). The average age of diagnosis was 33.0 ± 12.7 years in the LOTS participants and 51.5 ± 6.6 years in the LOSD participants (Fig. 1B), indicating there is an average diagnostic delay of 19.2 years and 33.3 years, from the onset of symptoms, respectively. The large range of age at diagnosis in LOTS participants (0–67 years) is due in part to one participant who was diagnosed in utero via prenatal screening. LOTS participants (24.8 ± 8.8 years) required earlier use of handrails to climb stairs compared to LOSD participants (29.5 ± 12.2 years); however, two LOTS participants (aged 33 and 41) did not require handrails at the time of evaluation (Fig. 1C). On average, gait impairment (Fig. 1D) was first noticed earlier in the LOTS participants (22.0 ± 7.6 years) than in the LOSD participants (36.4 ± 4.7 years); however, five LOTS participants and 1 LOSD participant indicated they were “not sure” when they first experienced impaired gait.

### Genotype

Fifteen of the 21 LOTS participants (71.4%) had one allele with the c.805G > A (p.G269S) variant, the most common variant associated with LOTS. An additional 3 participants (15.3%) were homozygous for this mutation.

Likely causative variants were identified in *HEXA* and *HEXB* in all individuals enrolled in the study and classified according to the 2015 Richards ACMG guidelines [60] and the updated recommendations from the ACMG Sequence Variant Interpretation Working Group [61–65]. Most participants were compound heterozygous for pathogenic (PATH) or likely pathogenic (LPATH) variants. In total, 16 variants were identified and classified; 9 in *HEXA* and 7 in *HEXB*; 13 were classified as PATH and 3 as LPATH (see Supplemental Table 2). These classifications align well with previous ClinVar entries, with the addition of one previously unseen LPATH variant as well as one large multi-exon deletion. Two participants (brothers) with the same nonsense HEXB variant did not have a variant in trans. Numerous functional studies support decreased activity in all 5 of the missense variants classified [66–75].

### Functional testing

TGUG (Fig. 1E) performance was similar in LOTS (17.05 ± 10.64 s) and LOSD (16.87 ± 9.042 s). However, these values were higher than the normative; healthy adults between 20–59 years achieve mean TGUG scores ranging from 8.57–9.90 s [76] For the 9HP test (Fig. 1F) on the dominant hand, LOTS participants had slightly higher values on average with greater variability (33.18 ± 16.82 s) compared to LOSD participants (27.41 ± 7.412 s). Comparatively, normative data for the 9HP test shows 20–74-year-olds ranging between 16.1–22.0 s [77] LOTS participants also tended to score higher on TMT-A (27.52 ± 10.41 s) than LOSD participants (21.31 ± 3.486 s, Fig. 1G). These values generally fell within normative data, with reports of mean scores for TMT-A in 18–74-year-olds ranging between 22.93 ± 6.87 s and 40.13 ± 14.48 s. [78] Results for TMT-B (Fig. 1H) were also higher in LOTS participants (63.14 ± 28.90 s) than LOSD participants (45.14 ± 16.52 s). Here, too, the LOTS and LOSD data fell within normative values, with mean values for TMT-B in 18–74-year-olds ranging between 48.97 ± 12.69 s and 86.27 ± 24.07 s [78].

### Ataxia

**Fig. 2:**
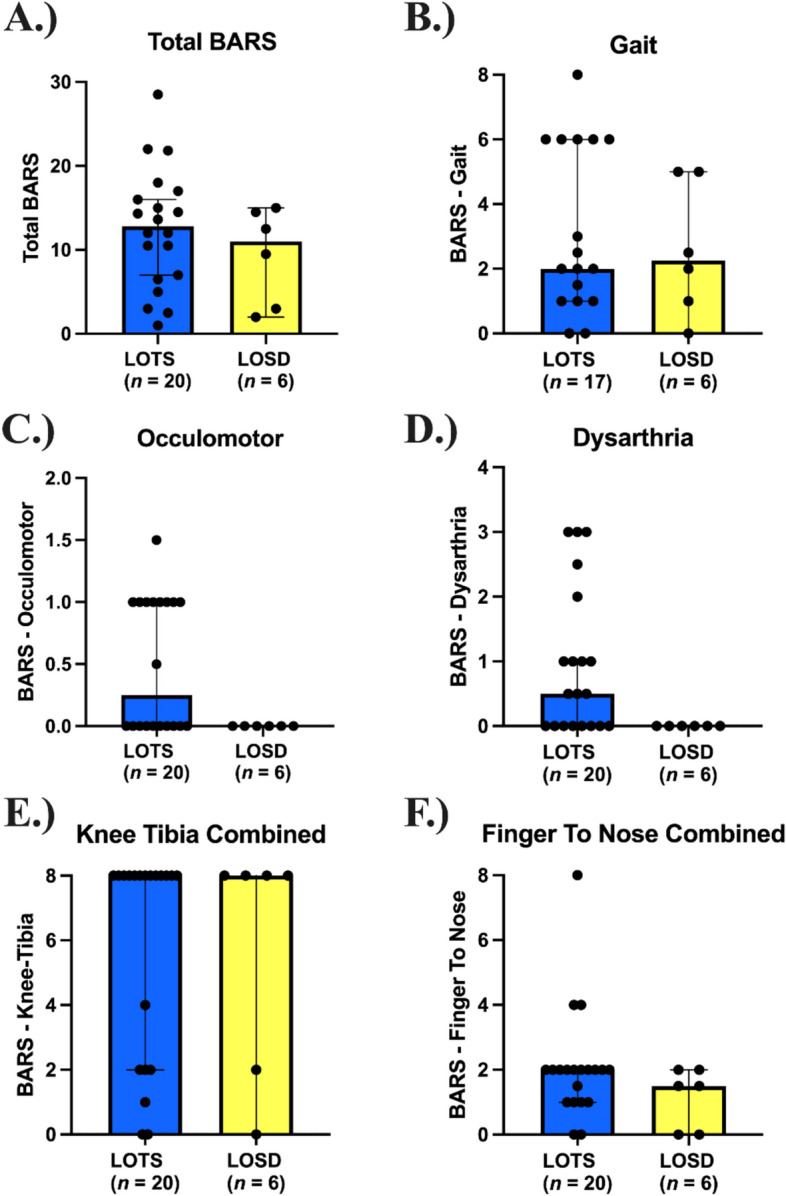
Brief Ataxia Rating Scale (BARS) Scores. LOTS participants are shown in blue and LOSD participants are shown in yellow. **A** Total BARS score out of 30. **B** BARS Gait Subdomain. **C** BARS Oculomotor Subdomain. **D** BARS Dysarthria Subdomain. **E** BARS Knee Tibia Test (total of bilateral measurements). F. BARS Finger-to-nose test (total of bilateral measurements). Total BARS scores were scaled to 30 for individuals who were unable to ambulate and complete BARS Gait testing

Total BARS scores (out of 30, Fig. 2A) were similar in LOTS (12.8 [IQR: 6.6, 16.75]) compared to LOSD (11.0 [IQR: 2.8, 14.6]). BARS finger-to-nose domain scores (out of 8, Fig. 2F) were higher in LOTS (2.0 [IQR: 1.0, 2.0]) compared to LOSD (1.5 [IQR: 0, 2.0]), which was skewed as one LOTS participant with severe dysmetria scored an 8. BARS oculomotor domain scores (out of 2, Fig. 2C) were higher in LOTS (0.25 [IQR: 0, 1.0]), compared to LOSD as all 6 LOSD individuals scored a 0, implying normal eye movement. BARS dysarthria scores (out of 4, Fig. 2D) were higher in LOTS (0.5 [IQR: 0, 1.8]), compared to LOSD as all 6 participants with LOSD scored a 0 (normal speech). BARS knee-tibia scores (out of 8, Fig. 2E) were similar between LOTS (8.0 [IQR: 2.0, 8.0]), and LOSD (8.0 [IQR: 1.5, 8.0]). BARS gait scores (Fig. 2B) were also similar between LOTS (2.0 [IQR: 1.0, 6.0]) and LOSD (2.3 [IQR: 0.8, 5.0]). BARS knee-tibia scores and gait are substantially impacted by the associated severe lower extremity weakness in participants, precluding completion of a task that did not necessarily indicate ataxia.

### Gait analysis

**Fig. 3:**
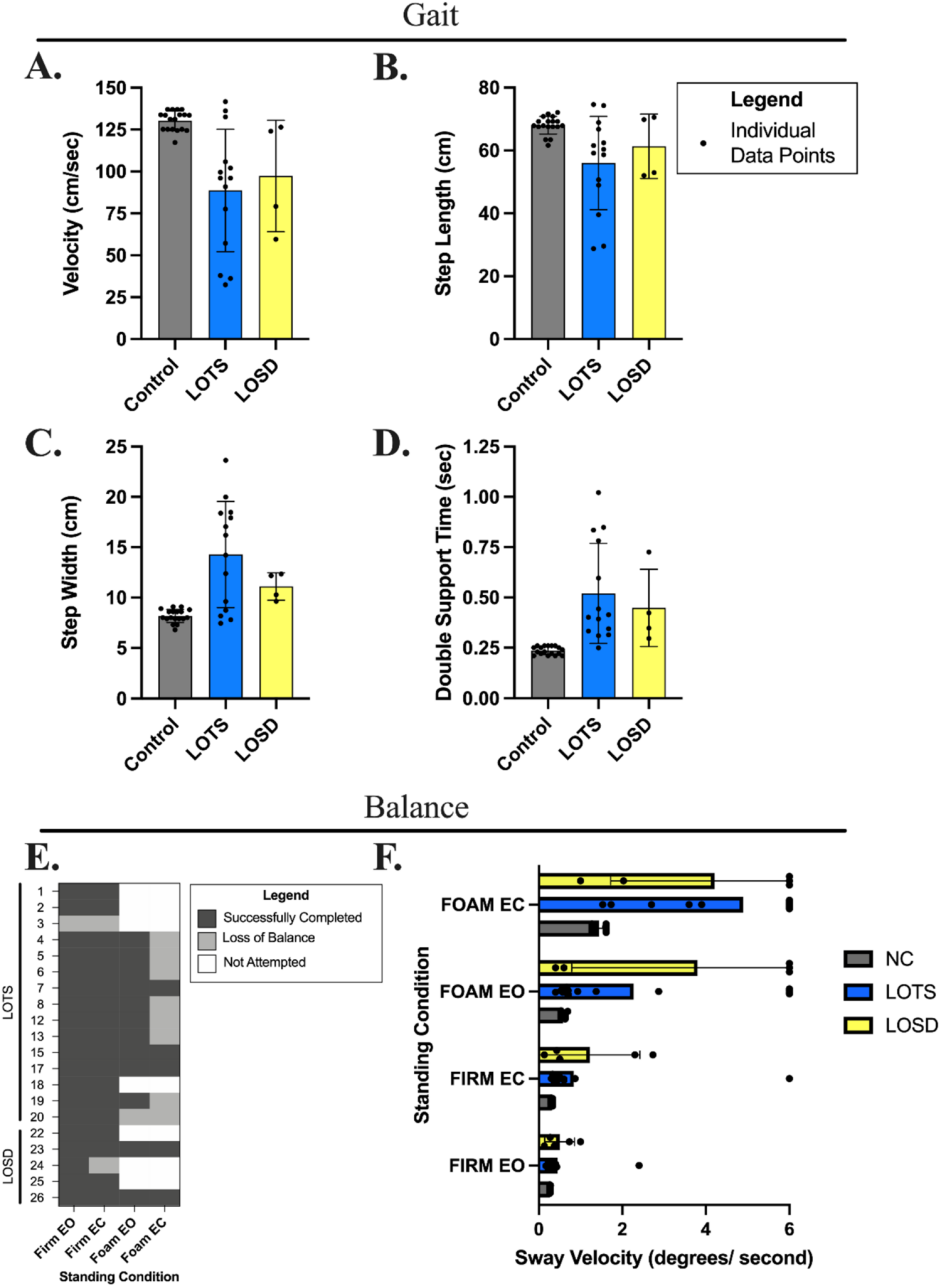
Gait and balance testing results. Gait parameters recorded for LOTS (blue), LOSD (yellow), and their age/sex matched controls (grey). **A** Gait velocity (cm/s), **B** step length (cm), **C** step width (cm), and **D** double support time (sec) are shown for each group. Data are presented as individual data points with the group mean ± SD, *n* = 15 LOTS (9 female, 6 male), *n* = 5 LOSD (2 female, 3 male), *n* = 18 control (10 female, 8 male). **E** mCTSIB Balance test completion per condition displayed by cohort (LOTS and LOSD) and participant number (Table 1). Participants who successfully completed all 3 trials for that condition are shown in dark gray, participants who lost balance during at least one trial are shown in light gray, and participants who did not attempt the testing are shown in white. **F** mCTSIB sway velocity of LOTS participants, LOSD participants, and their age/sex matched controls per condition in order of difficulty (top to bottom). Data are represented as individual data points with the group mean marked as a diamond (blue for LOTS, yellow for LOSD, and grey for controls). Foam EC = standing on foam surface with eyes closed, Foam EO = standing on foam surface with eyes open, Firm EC = standing on firm surface with eyes closed, Firm EO = standing on firm surface with eyes open

Fourteen LOTS and 4 LOSD participants were tested alongside eighteen age- and sex-matched controls (one control per participant) from MacKay et al. [41] As shown, abnormalities were identified for all gait parameters (Fig. 3A-3D). As a group, LOTS and LOSD participants (88.73 ± 36.49 cm/s and 97.31 ± 33.24 cm/s, respectively) walked slower than controls (130.34 ± 5.87 cm/s). LOTS participants had a shorter step length than controls (56.02 ± 14.83 cm vs. 68.03 ± 2.82 cm), but the difference was less prominent in the LOSD cohort (61.34 ± 10.24 cm vs. 68.03 ± 2.82 cm). Additionally, both LOTS and LOSD participants had a wider base of support than controls, as evident by step width (LOTS: 14.29 ± 5.27 cm; LOSD: 11.11 ± 1.36 cm respectively; Controls: 8.18 ± 0.65 cm, and both spent longer time in the double support phase of gait (LOTS: 0.52 ± 0.25 s; LOSD: 0.49 ± 0.19 s; Controls: 0.24 ± 0.02 s).

### Balance testing

Fifteen LOTS and 5 LOSD participants attempted balance testing, with relatively few participants completing all trials without losing their balance (3 LOTS and 2 LOSD participants, Fig. 3E).

### Sway velocity—firm condition

Most participants were able to stand on the firm surface with eyes open or closed without losing their balance (Fig. 3E). Sway velocity with eyes open was similar between the LOTS (0.45 ± 0.55 degrees/sec), LOSD (0.50 ± 0.36 degrees/sec), and control (0.27 ± 0.01 degrees/sec) groups,

although one severely impaired individual in the LOTS group had a particularly high score (Fig. 3F). In the eyes closed condition, average sway velocity was higher for the LOTS (0.83 ± 1.44 degrees/sec) and LOSD (1.22 ± 1.20 degrees/sec) cohorts than controls (0.31 ± 0.02 degrees/sec), but the results were skewed by a few individuals.

### Sway velocity—foam condition

Only 12 participants (10 LOTS and 2 LOSD) were able to stand on the foam surface with their eyes open, and only 5 participants (3 LOTS and 2 LOSD) were able to stand on the foam surface with their eyes closed (Fig. 3E). Sway velocity was higher in the eyes open condition in both the LOTS (2.27 ± 2.40 degrees/sec) and LOSD (3.80 ± 3.01 degrees/sec) compared to controls (0.58 ± 0.06 degrees/sec), however this was skewed by the subset of individuals (5/21 LOTS [23%] and 3/6 LOSD [50%]) unable to successfully complete the testing. Similarly in the eyes closed condition, LOTS (4.90 ± 1.71 degrees/sec) and LOSD (4.21 ± 2.48 degrees/sec) exhibited higher sway velocities compared to controls (1.44 ± 0.17 degrees/sec).

### Dynamometry quadriceps and hamstring strength testing

**Fig. 4:**
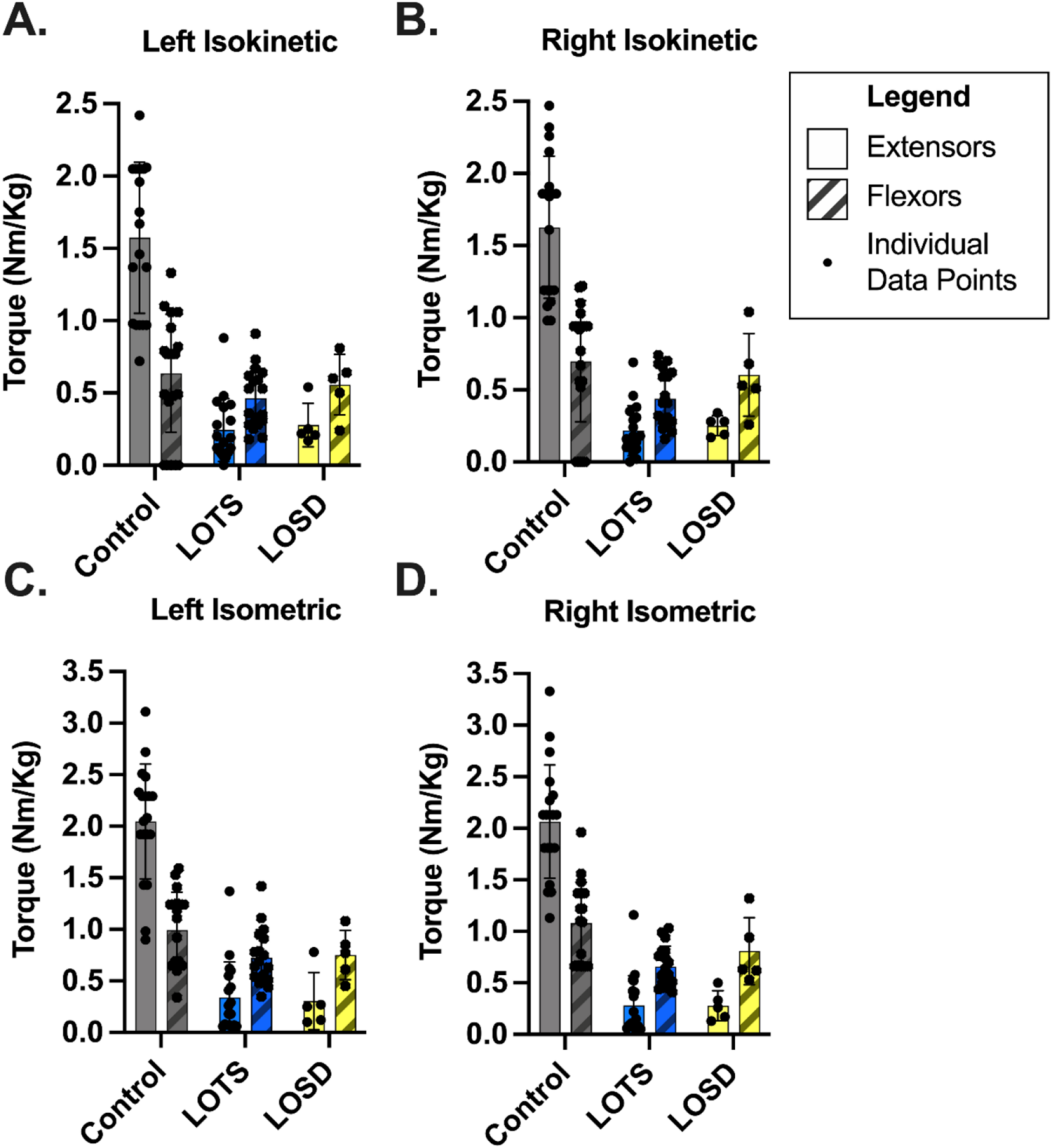
Knee extensors (solid fill) and flexors (striped fill) strength in LOTS (blue) and LOSD participants (shown in yellow), and their age/sex matched controls (shown in grey). **A** Left isokinetic, **B** right isokinetic, **C** left isometric, and **D** right isometric torque normalized to the participant’s weight (Nm/kg) are shown for each group. Data are presented as individual data points with the group mean ± SD, *n* = 18 LOTS (11 female, 7 male), *n* = 5 LOSD (2 female, 3 male), *n* = 19 control (10 female, 9 male). Age/sex matched controls were not available for 3 LOTS participants and 1 LOSD participant

Eighteen LOTS, 5 LOSD, and 19 control participants completed strength testing. There was no qualitative difference between left and right isokinetic torques or between left and right isometric torques within any of the three groups (Fig. 4A-D). Isokinetic and isometric extension was qualitatively larger in the control group (left isokinetic: 1.5 ± 0.5 Nm/kg; left isometric: 2.0 ± 0.5 Nm/kg) than in the LOTS (left isokinetic: 0.25 ± 0.2 Nm/kg; left isometric: 0.25 ± 0.25 Nm/kg) and LOSD (left isokinetic: 0.25 ± 0.2 Nm/kg; left isometric: 0.25 ± 0.25 Nm/kg) groups. However, there was no qualitative difference in isokinetic and isometric flexion between the control (left isokinetic: 0.7 ± 0.3 Nm/kg; left isometric: 0.9 ± 0.5 Nm/kg), LOTS (left isokinetic: 0.4 ± 0.2 Nm/kg; left isometric: 0.7 ± 0.3 Nm/kg), and LOSD (left isokinetic: 0.6 ± 0.2 Nm/kg; left isometric: 0.7 ± 0.3 Nm/kg) groups. Control group isokinetic extension (~ 1.5 Nm/kg,) was approximately twice as large as isokinetic flexion (~ 0.7 Nm/kg). Conversely, LOTS isokinetic extension (~ 0.25 Nm/kg) was approximately half as large as isokinetic flexion (~ 0.4 Nm/kg). In the LOSD group, isokinetic extension (~ 0.25 Nm/kg) was also approximately half as large as isokinetic flexion (~ 0.5 Nm/kg).

### Sensory examination and electrodiagnostic testing

All 27 participants completed time-to-extinction of vibration perception. Vibration perception was absent in 67% (4/6) of LOSD participants with the remaining 2 LOSD participants both scoring severely decreased. Vibration perception was normal in 71% (15/21) of LOTS participants, mildly decreased in 19% of LOTS participants, and severely decreased in the remaining 10% (2/21) of LOTS participants (Supplement Table 1).

**Fig. 5:**
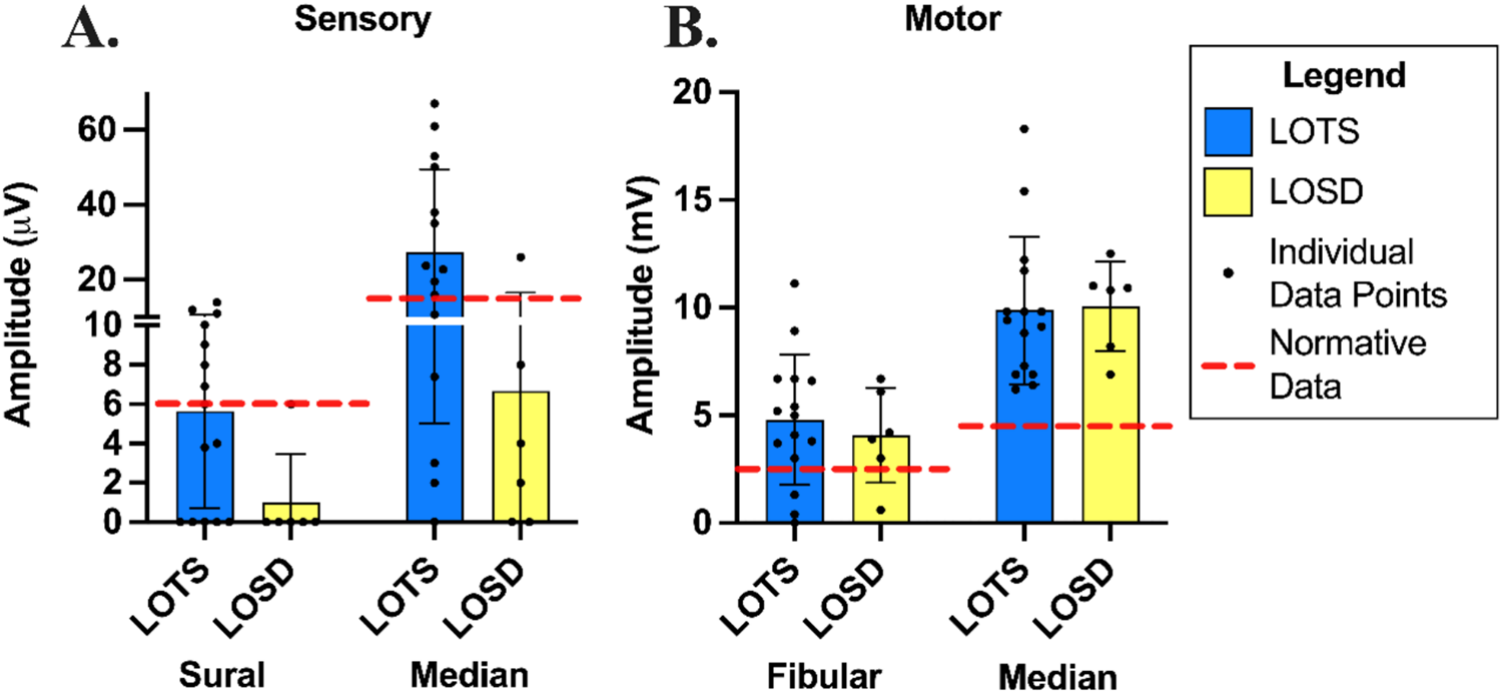
Electrodiagnostic amplitudes of A. sensory nerves (SN) and B. motor nerves (MN) recorded for LOTS and LOSD participants. LOTS participants, *n* = 15 (10 female, 5 male), are shown in blue and LOSD participants, *n* = 6 (3 women, 3 male) are shown in yellow. [Normal values: sural SN ≥ 6.0 µV, median SN ≥ 15.0 µV, fibular MN ≥ 2.5 mV, median MN ≥ 4.5 mV (shown in red dashed line).]

Fifteen LOTS and 6 LOSD participants completed electrodiagnostic testing. Sural and median sensory amplitudes were lower in the LOSD (sural: 1.0 ± 2.5 μV, median: 6.7 ± 9.9 μV) group compared to LOTS (sural: 5.6 ± 4.9 μV, median: 27.3 ± 22.2 μV) and the lower limit of normative data (sural: 6 μV, median: 15 μV Fig. 5A), respectively. The fibular and median motor amplitudes were not different between the LOSD (fibular: 4.1 ± 2.2 mV, median: 10.1 ± 2.1 mV) and LOTS (fibular: 4.8 ± 3.0 mV, median: 9.9 ± 3.4 mV) cohorts, and both were above the upper limit of normal (fibular: 2.5 mV, median: 4.5 mV Fig. 5B).

Sural Sensory Nerve Action Potentials (SNAPs) were absent in 83% of LOSD (5/6). The remaining LOSD participants had an amplitude at the normal amplitude threshold (6 µV). This contrasts with 33% of LOTS (5/15) with absent sural nerve SNAPs, 2 (13%) with low amplitude SNAPs and 7 (45%) with normal sural SNAPs. Similarly, all LOSD participants had reduced (4/5), or absent 1/5 median nerve SNAP amplitude. The mean median nerve SNAP amplitude in LOSD was 4.0 µV (compared to normal laboratory control threshold of ≥ 15 µV), whereas LOTS participants’ mean median nerve SNAP amplitude was 23.7 µV, with 9/15 participants (60%) having normal amplitudes, one (6%) with absent amplitude, and 5/15 (33%) with below-normal amplitude. The motor nerve conduction studies revealed fewer abnormalities than the sensory nerve conduction studies. Compound Motor Action Potential (CMAP) amplitudes in the fibular nerve recording from the extensor digitorum brevis muscle were low in 3/15 (20%) participants with LOTS and in 1/5 with LOSD (20%). Median nerve CMAP amplitudes were normal in both participant group.

Both LOTS and LOSD participants had active denervation with the presence of fibrillations, positive sharp wave and/or complex repetitive discharges. Both groups also showed chronic neurogenic changes with the presence large motor units signifying reinnervation on needle EMG, particularly in the triceps, quadriceps, medial gastrocnemius, and tibialis anterior muscles, though neurogenic changes were slightly more prevalent in the LOTS subjects. These needle EMG findings are more consistent with a motor neuronopathy affecting particular muscle groups and correlating severe weakness on clinical exam rather than a length-dependent neuropathy.

### MRI analysis

**Fig. 6:**
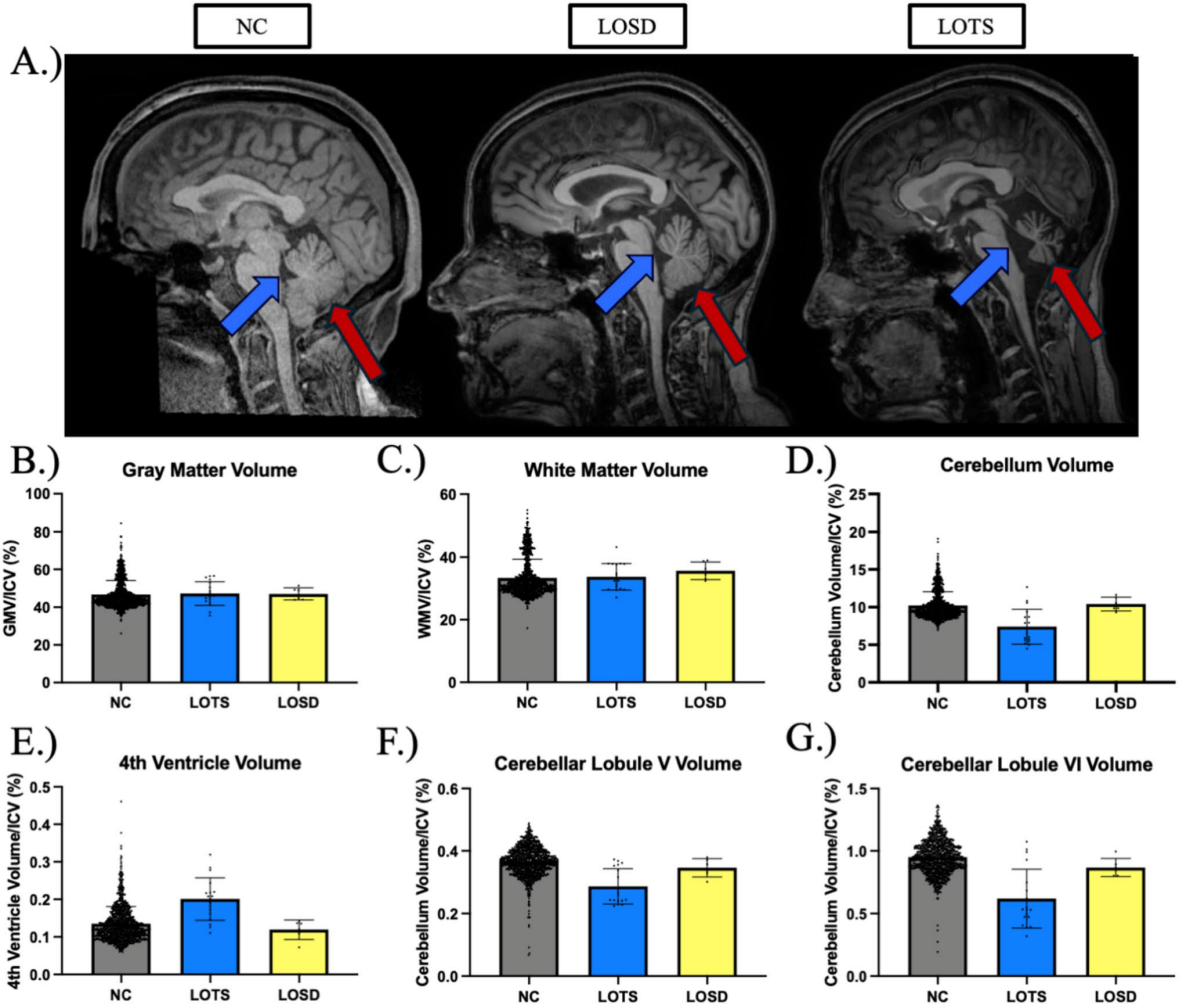
MRI Analysis. **A** Sagittal slice of one 41-year-old female neurotypical control (NC), one 44-year-old female late-onset Sandhoff disease (LOSD) participant, and one 42-year-old female late-onset Tay-Sachs (LOTS) participants. The blue arrows designate the 4th ventricle, and the red arrows designate the cerebellum of the three participants. The LOTS participant demonstrates considerable 4th ventricle enlargement and cerebellar atrophy compared to both the LOSD participant and NC. **B** Volumetric analysis of gray matter volume (GMV) between NC, LOTS, and LOSD participants. **C** Volumetric analysis of white matter volume (WMV) between NC, LOTS, and LOSD participants. **D** Volumetric analysis of cerebellum volume between NC, LOTS, and LOSD participants. **E** Volumetric analysis of 4th ventricle volume between NC, LOTS, and LOSD participants. **F** Volumetric analysis of cerebellar lobule V and **G** lobule VI between LOTS and LOSD. All volumes were controlled for total intracranial volume (ICV). LOTS participants are shown in blue (*n* = 18), LOSD participants are shown in yellow (*n* = 6), and neurotypical controls are shown in gray (*n* = 1038)

18 LOTS and 6 LOSD participants had MRI testing at the cross-sectional evaluation. Gray matter volume (GMV) was similar between NC (46.8 ± 7.3 percent of ICV), LOTS (47.2 ± 6.1 percent of ICV), and LOSD (46.6 ± 3.4 percent of ICV, Fig. 6B). White matter volume (WMV) was similar between NC (33.3 ± 6.0 percent of ICV), LOTS (33.6 ± 4.1 percent of ICV), and LOSD (36.3 ± 2.6 percent of ICV, Fig. 6C). Cerebellar volumes were lower in LOTS (7.6 ± 2.3 percent of ICV) compared to both NC (10.2 ± 1.8 percent of ICV) and LOSD (10.5 ± 1.0 percent of ICV, Fig. 6D). Conversely, 4th ventricle volume was higher in LOTS (0.20 ± 0.06 percent of ICV) compared to both NC (0.13 ± 0.05 percent of ICV) and LOSD (0.12 ± 0.03 percent of ICV, Fig. 6E). When analyzed at the lobule level (Figs. 6F, G), LOTS participants showed decreased volumes of both lobule V and VI (0.29 ± 0.06, 0.62 ± 0.24) compared to LOSD (0.34 ± 0.03, 0.84 ± 0.04) and NC (0.37 ± 0.05, 0.95 ± 0.14).

## Discussion

**Fig. 7:**
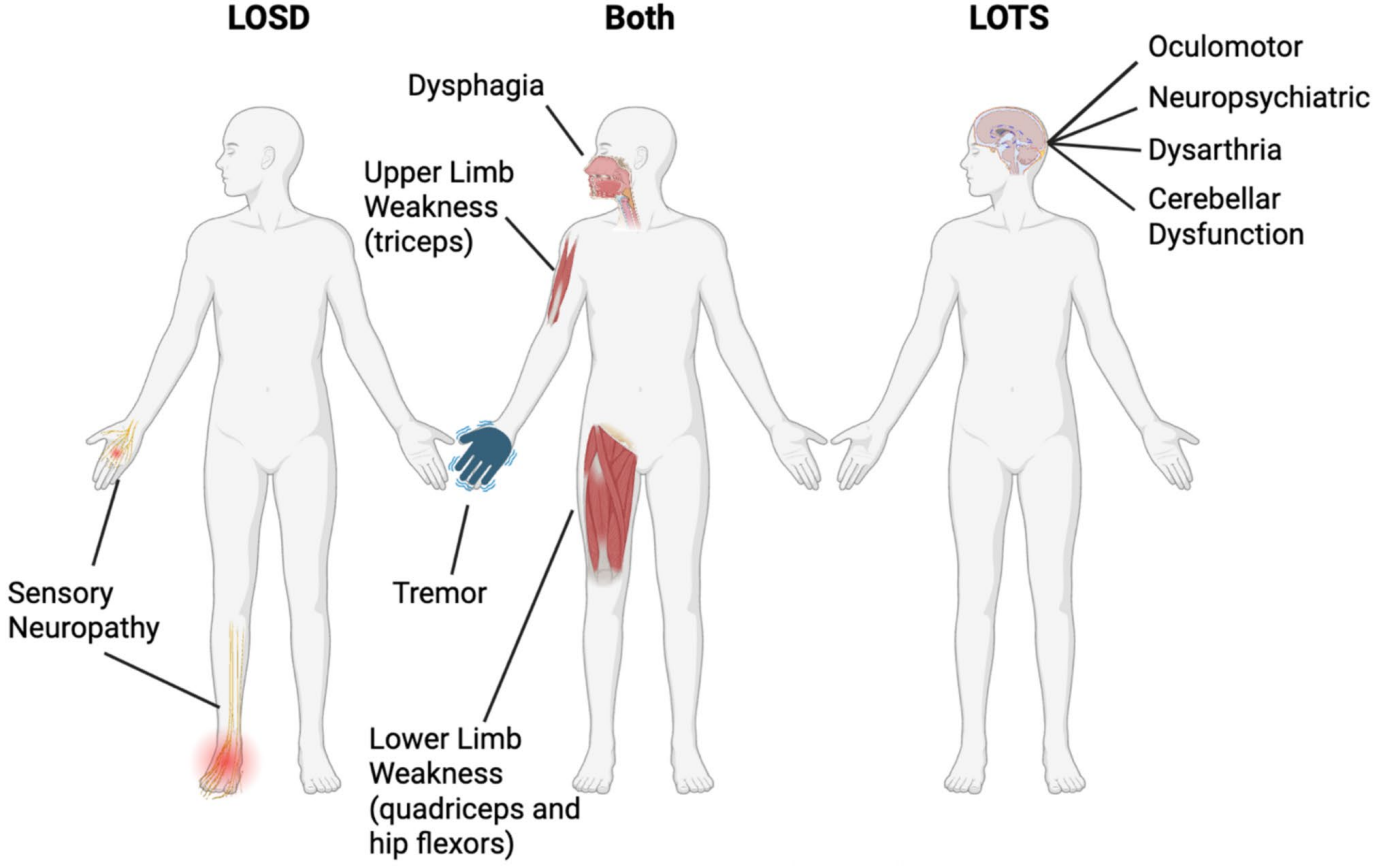
Illustration of symptom similarities and differences between late-onset GM2 gangliosidoses (both), late-onset Tay-Sachs (LOTS), and late-onset Sandhoff disease (LOSD)

Despite shared biochemical deficiencies of ß-hexosaminidase A and significant clinical overlap, differences have emerged between late-onset Tay-Sachs (LOTS) and Sandhoff diseases (LOSD), and this study aimed to further characterize this dichotomy through the cross-sectional phenotypic evaluations of 21 participants with LOTS and 6 participants with LOSD. Clinically, we found both LOTS and LOSD to frequently share lower limb weakness later progressing to upper limb weakness (Fig. 7). LOTS participants predominantly had signs related to cerebellar dysfunction including cerebellar ataxia, dysarthria, and oculomotor findings as well as a higher prevalence of psychiatric symptoms, which were not found in the LOSD patients. In the LOSD patient group, we saw a more severe length dependent sensory neuropathy which was not present in LOTS and contributed to a picture of proprioceptive ataxia.

In a natural history study by Masingue et al. [17] describing 57 adult participants with GM2 gangliosidosis, three main clinical presentations, or some combination of the three, were identified: lower limb weakness progressing to upper limb weakness, cerebellar ataxia, and psychiatric symptoms (only in LOTS) [17]. The 26 GM2 gangliosidosis participants described in this study are similar, as the probands all fit into one of the ascribed categories. Lower limb weakness was highly prevalent in both LOTS and LOSD as evidenced by gait disturbances, strength testing, balance testing, presenting symptom(s), and the high frequency of participants requiring handrails to climb stairs. Upper limb weakness primarily affecting the triceps muscles was also highly prevalent in both LOTS and LOSD, occurring later in the disease course.

Isokinetic and isometric strength of both the hamstrings and quadriceps was found to be impaired in both LOTS and LOSD, with the knee extensors being profoundly weak. In both LOTS and LOSD, average knee extension strength was roughly 20% of predicted when compared to age- and sex-matched controls. These results confirm our clinical observations, and reports in the literature, of knee extensors being more affected by both disease processes than knee flexor muscle groups [10, 17]. The rectus femoris, a bi-articular muscle acts both as a knee extensor and as a hip flexor. Therefore, the observed hip flexor weakness in LOTS and LOSD is likely to be directly related, at least in part, to the observed profound atrophy and weakness in the rectus femoris muscle. Lower limb muscle strength including hip flexion and knee extension are crucial components for assuming an upright position, maintaining an upright stance, and controlling gait and balance, all of which were altered in LOTS and LOSD compared to normative controls.

Balance testing demonstrated increased sway velocities in the LOTS and LOSD cohorts compared to controls, particularly in the foam condition. During the testing, participants in both cohorts frequently locked their knees to maintain upright postural control and counterbalancing the quadriceps weakness during the mCTSIB balance test. In fact, many participants reported they could not rely on knee strength and were afraid if their knees buckled, they would collapse and could not prevent falling. Most participants were unable to properly execute the balance testing procedure due to lack of proximal antigravity strength to complete the task. This was particularly prevalent during the balance standing on a foam surface with eyes closed which highlights a contribution of proprioceptive loss, especially in patients with LOSD. Balance testing substantiated the findings of a complex interaction of lower extremity antigravity strength, cerebellar deficits and proprioceptive function leading to impaired balance function in LOTS and LOSD patients, prompting the need for novel approaches for evaluating balance, especially on patients at the most advanced stages of disease.

Ataxia, as assessed by BARS, showed similarities between LOTS and LOSD including upper and lower extremity scores for dysmetria (Fig. 2). However, similar to the balance results, there were difficulties parsing the exact contributions of different neurological deficits to the overall experience of imbalance and falls in our study population, especially weakness and cerebellar deficits in LOTS and weakness and proprioceptive deficits with proprioceptive ataxia in LOSD. However, the BARS highlighted two considerable disparities between LOTS and LOSD. The oculomotor portion of the BARS was abnormal in LOTS and normal in LOSD. Saccadic dysmetria has previously been described in LOTS [10, 79], and further demonstrated in a cohort including 7 LOTS participants but not in 3 LOSD participants [80]. Our study further supports the finding that oculomotor dysfunction is more prevalent in LOTS than LOSD. It is well known that cerebellar dysfunction affects the oculomotor system in many ways including saccadic intrusion, “square wave” jerks, saccadic dysmetria, abnormal VOR and disruption of normal saccade velocity [10]. Cerebellar lobule topologies in relation to oculomotor abnormalities localizes primarily to lobules V, VI and VII, and both Crus I and II [81,82].

Furthermore, speech articulation was also substantially altered in most (71%) LOTS participants and in none of the participants with LOSD. Progressively worsening dysarthria has been demonstrated in late-onset GM2 gangliosidosis [83], and isolated dysarthria has even been described as an initial clinical presentation in LOTS [21]. This study diverges from previous reports showing an absence of dysarthria in our LOSD participants while illustrating a wide spectrum of variability in LOTS from, participants with long disease course and entirely normal speech articulation to younger participants with profound intelligibility challenges. LOTS-associated dysarthria is characterized by distinct and complex features including abnormal articulation, explosive features and rapid pace, among others. Dysarthria in LOTS has been attributed to cerebellar dysfunction, and indeed, there is supporting evidence that dysfunction or injury to lobules V and VI are linked to dysarthric speech in other settings. [84]

Neuropsychiatric symptoms including the emergence of acute mania and psychosis, even as a presenting symptom, have long been known in LOTS [85–87]. Indeed, in this cross-sectional evaluation, 5/21 (24%) of the LOTS participants but none of the LOSD participants had a history of psychiatric symptoms; indeed, 2/16 (13%) of the LOTS probands in this study had psychosis as the presenting symptom. Our observation is consistent with Masingue et al. [17], where a combined cohort of 57 participants, including participants evaluated at their center and participants reported from other studies (40 LOTS and 17 LOSD), psychiatric symptoms were the initial presentations of LOTS for 11/40 (28%) participants, while none of the 17 LOSD participants presented with psychiatric symptoms. Only 1 LOSD participant developed psychiatric symptoms later in the disease course [17]. The basis for neuropsychiatric symptoms in LOTS and the apparent lower prevalence in LOSD is not fully understood, however, the finding of more profound cerebellar atrophy in LOTS vs. LOSD may provide a clue to these differences. There is growing evidence demonstrating that cerebellar dysfunction, and more specifically the posterior cerebellum, has an impact on behavior. The cerebellar cognitive affective syndrome (CCAS), encompassing several cognitive and behavioral deficits, is caused by damage to the posterior cerebellum (lobules VI, VII, and IX), and results in impaired executive function and affect regulation [88–90].

Magnetic resonance imaging (MRI) demonstrated expected results based on previous reports [26,27, 29, 59]. First, both supratentorial gray and white matter volumes were not influenced by LOTS or LOSD (Fig. 6). LOTS participants had reduced cerebellar volume (including lobules V and VI) and associated enlargement of the 4th ventricle. While the differential finding of cerebellar atrophy in LOTS vs. LOSD has been described in prior scoping review [28], this finding, including its detailed the cerebellar lobule topology, has not been extensively quantified volumetrically until recently [59]. The discrepancy in both cerebellar atrophy and psychiatric and speech manifestations in LOTS versus LOSD imply that progressive severe cerebellar involvement earlier in life or perhaps, even prenatally, contribute to both these clinical findings.

Analysis of the individual cerebellar lobules has been described in LOTS [29, 59], demonstrating atrophy in the posterior cerebellum. However, the study by Májovská et al. [29], found no relationship between cerebellar lobule atrophy and presence of psychiatric symptoms. However, their analysis included only seven participants with psychiatric impairment, and the participants they include in their analysis had a heterogeneous group of psychiatric symptoms including depression, anxiety, adjustment disorder, and cognitive deficits [29]. This may indicate that the relationship between neuropsychiatric symptoms and cerebellar atrophy is not direct, and that multiple cerebellar associated clinical features (dysarthria, oculomotor dysfunction, psychiatric impairment, and cerebellar ataxia) originate from cerebellar atrophy. More detailed phenotyping including detailed neuropsychiatric analysis and MRI-derived cerebellar lobule analysis including cerebellar white matter diffusion tensor imaging is needed [27].

Clinical and electrodiagnostic studies in our cohort present an additional element of the LOTS versus LOSD phenotypic dichotomy. While both LOTS and LOSD participants appear to experience identical patterns and severity of lower motor neuronopathy with predilection for hip flexors, knee extensors, and triceps muscles, LOSD but not LOTS participants showed substantial and disabling sensory deficits affecting all sensory modalities (proprioception, vibration, temperature and pinprick) and reflected in symptoms of dysautonomia including but not limited to reported symptoms of chronic diarrhea, thermoregulatory deficits, and heat-related acro-paresthesia, present in all of our LOSD participants. Severe proprioceptive deficits in LOSD further contribute to their sense of imbalance. While modest sensory NCV deficits have been reported in a prior LOTS study, LOTS participants are seldom symptomatic [17].

This study, although one of the largest reported single cohorts of late-onset GM2 participants, has several limitations. Principally, this study is limited by the sample size (*n* = 27). This highlights the rarity of the disease and likely both its under- and delayed diagnosis. This study is also limited by incomplete data collection due to personal choice or safety concerns. Furthermore, we scored ataxia using the BARS scale by a single rater, therefore, interrater reliability was not established. The late-onset GM2 gangliosidoses are much more than an ataxic syndrome. Further efforts should be undertaken to develop disease specific scales that include neuromuscular weakness, dysarthria, cerebellar motor and cognitive deficits (CCAS), and sensory dysfunction. Participant- and caregiver- related outcomes in relation to disease experience should be incorporated as a part of this effort [30]. The normative controls for MRI analysis were scanned using different scanners and acquisition protocols, a limitation of the MRI results, however all the acquisition protocols were of high resolution (1 mm [3] isotropic voxels or better), similar to the study participants.

In conclusion, this study highlights key differences between LOTS and LOSD including cerebellar dysfunction, imaging abnormalities, and sensory neuropathy. LOTS participants showed increased prevalence and severity of dysarthric speech, oculomotor abnormalities, and neuropsychiatric symptoms, all of which can relate to cerebellar dysfunction. In contrast, LOSD participants experienced sensory deficits, not present in the participants with LOTS. Future studies are needed to understand the genetic and molecular explanation for the differences between LOTS and LOSD despite a shared enzymatic defect.

## Supplementary Information

Below is the link to the electronic supplementary material.

Supplementary file1 (XLSX 14 KB)

Supplementary file2 (XLSX 21 KB)

## Data Availability

The GM2 data described in this manuscript are available from the corresponding author upon reasonable request. Neurotypical control MRI data are available from OpenNeuro at the following links; NIMH :10.18112/openneuro.ds004215.v1.0.0[49,50] Neurocognitive Aging Data Release with Behavioral, Structural, and Multi-echo functional MRI measures: https://openneuro.org/datasets/ds003592/versions/1.0.13 [51,52], Paingen_Placebo: https://openneuro.org/datasets/ds004746/versions/1.0.1 [53,54], AgeRisk: https://openneuro.org/datasets/ds004711/versions/1.0.0 [55,56].
